# Severe Eosinophilic Syndrome: Highly Unlikely Associated with the Use of Probiotic Supplements!

**DOI:** 10.1155/2013/769127

**Published:** 2013-03-17

**Authors:** Arthur C. Ouwehand, Ger T. Rijkers

**Affiliations:** ^1^Active Nutrition, DuPont Nutrition and Health, 02460 Kantvik, Finland; ^2^Department of Science, University College Roosevelt, 4330 AB Middelburg, The Netherlands

## Abstract

A recent report in this journal suggested a causal relation between probiotic consumption and eosinophilia. In our opinion, the data presented does not suggest such a relationship. The two described eosinophilia cases have not been shown to be caused by infection and certainly not by probiotic infection. The consumed probiotics could not be retrieved in shops, so their identity remains unknown. Furthermore, the alleged consumption took place 2–4 weeks prior to the onset of the symptoms; during such time period, probiotics tend to have disappeared from the intestine. Because most probiotic health benefits are strain specific, also potential risks are strain specific. Thus, generalizing a risk to probiotics as a class is incorrect. We do, however, agree with the authors of the case report that quality control of probiotics should be rigorous. We also do not dispute that there may be certain risk groups (e.g. severely immune-compromised patients), where probiotic use should be carefully monitored. In conclusion, the data presented in the case report do not indicate that specific probiotics strains might cause eosinophilia.

## 1. Case

With interest and concern did we read the work of Mendoza and coworkers [1] describing two cases of severe eosinophilic syndrome temporally associated with the use of a nonspecified brand of probiotics. The authors conclude that the eosinophilic syndrome is likely to be associated with the consumption of probiotics. We would like to raise serious concerns with this line of reasoning and the suggestions made in the paper.

Peripheral eosinophilia can have various causes, among others bacterial infection. The authors do, however, not report on observing any infection. This is very unfortunate as the characterisation of an infectious agent would have allowed for the unequivocal identification of the cause of the observed eosinophilia. In its absence, other aetiologies cannot be ruled out.

The suggestion that the reported cases of eosinophilia were caused by probiotics is based on the recollection of the subjects that they consumed such products two to four weeks prior to onset of the symptoms. Obviously, this is a very weak basis to say the least; the patients will also recall other things they did or did not do few weeks before their disease. In the absence of any physical proof of the products consumed, not even a formal demonstration that both had used the same product, it is not certain that the patients actually did so. It is therefore well possible that any other food or drink they consumed may have been the cause. Even if they did consume probiotics, there is no solid indication that the consumed probiotics were associated with the eosinophilia, let alone that they would be the cause. During the EHEC outbreak in Germany in 2011, a wide range of vegetables (tomatoes, cucumbers, etc.) was indicated as potentially contaminated before sprouts turned out to be the cause [2]. Food safety is of utmost importance to protect consumers, and this applies for any type of food, including food supplements. In the patients described by Mendoza et al., the time span between the presumed consumption of probiotics and the onset of symptoms was 2–4 weeks, which actually suggests that probiotics are unlikely to be the cause. Probiotics colonize the gastrointestinal tract only transiently. Once consumption of probiotics is stopped, they generally disappear from the intestinal tract within a week or two [3]. Finally, the suggestion that “probiotics” (as a group) pose a risk for eosinophilic syndrome is a sweeping generalization. Probiotics are members of a very wide range of species and genera, ranging from bacteria to yeasts, and within these species, multiple strains exist with shared but also specific properties. It is therefore incorrect to state that “probiotics” have certain health benefits, and it is equally incorrect to state that “probiotics” pose a specific risk. Health benefits, and also risks, are strain specific although certain health benefits, and risks, can be shared by multiple strains.

Notwithstanding the above, we agree with the authors that the quality of probiotic preparations and food supplements in general should be checked to assure the highest possible standards. It is furthermore generally acknowledged that certain risk populations for the use of probiotics exist, mainly the immune compromised. Probiotics are, by definition, live microorganisms and may thus cause infection. In this respect, it is important to recognize the difference between strains from species that are commonly used as probiotics but originate from the hosts own microbiota and probiotics. An infection by, for example, *Lactobacillus* or *Saccharomyces* does not always have to be a probiotic infection. Strain identification is thus crucial for identifying probiotic infections.

In conclusion, in both cases which have been presented, it has not been established that an infection would have caused the eosinophilia, let alone that it would have been caused by a probiotic. The suggestion of a link with probiotic consumption is therefore based on speculation. 
